# Attentional gain is modulated by probabilistic feature expectations in a spatial cueing task: ERP evidence

**DOI:** 10.1038/s41598-017-18347-1

**Published:** 2018-01-08

**Authors:** Anna Marzecová, Antonio Schettino, Andreas Widmann, Iria SanMiguel, Sonja A. Kotz, Erich Schröger

**Affiliations:** 1 Institute of Psychology, Leipzig University, Leipzig, Germany; 2 Department of Experimental and Applied Psychology, Vrije Universiteit Brussel, Brussels, Belgium; 3 Department of Experimental-Clinical and Health Psychology, Ghent University, Ghent, Belgium; 4 Brainlab-Cognitive Neuroscience Research Group, Department of Clinical Psychology and Psychobiology, University of Barcelona, Barcelona, Spain; 5 Institute of Neurosciences, University of Barcelona, Barcelona, Spain; 6 Institut de Recerca Sant Joan de Déu (IR-SJD), Barcelona, Spain; 7 Faculty of Psychology and Neuroscience, Department of Neuropsychology & Psychopharmacology, Maastricht University, Maastricht, The Netherlands

## Abstract

Several theoretical and empirical studies suggest that attention and perceptual expectations influence perception in an interactive manner, whereby attentional gain is enhanced for predicted stimuli. The current study assessed whether attention and perceptual expectations interface when they are fully orthogonal, i.e., each of them relates to different stimulus features. We used a spatial cueing task with block-wise spatial attention cues that directed attention to either left or right visual field, in which Gabor gratings of either predicted (more likely) or unpredicted (less likely) orientation were presented. The lateralised posterior N1pc component was additively influenced by attention and perceptual expectations. Bayesian analysis showed no reliable evidence for the interactive effect of attention and expectations on the N1pc amplitude. However, attention and perceptual expectations interactively influenced the frontally distributed anterior N1 component (N1a). The attention effect (i.e., enhanced N1a amplitude in the attended compared to the unattended condition) was observed only for the gratings of predicted orientation, but not in the unpredicted condition. These findings suggest that attention and perceptual expectations interactively influence visual processing within 200 ms after stimulus onset and such joint influence may lead to enhanced endogenous attentional control in the dorsal fronto-parietal attention network.

## Introduction

Attentional and perceptual expectations are understood as mechanisms that facilitate perceptual processing. Attentional selection may be defined as a mechanism driven by information about behavioural relevance, while perceptual expectations are thought to capitalise on information about prior probability^1,2^. It has recently been proposed that attention and expectation dissociate in their influence on behavioural performance^3–5^. While attention increases detection sensitivity^6–8^, perceptual expectations are hypothesised to influence the response criterion, leading to a response bias^3,4,9^. The current study addressed how attention and perceptual expectations dissociate in their electrophysiological signatures, and how they may interact to optimise perception.

## Neural signatures of visuospatial attention

Neural signatures of attention have predominantly been studied with spatial cueing task^10^, in which stimuli are presented in the left or right visual field (LVF/RVF). Attention is directed to a relevant location by a cue either in a transient or in a sustained fashion^11,12^. Enhancements of early visual event-related potentials (ERP) in response to stimuli at attended compared to unattended locations have been interpreted as reflecting sensory gain for attended stimuli^13,14^. A modulation of the P1 component, the first positive deflection with a peak around 100–130 ms over lateral posterior electrode-sites, has been attributed to inhibitory processes in task-relevant and task-irrelevant neural structures^15,16^. On the other hand, an enhancement of the N1 component (peaking around 150–200 ms) in response to stimuli at attended locations has been interpreted as a facilitatory mechanism of attentional selection^17,18^, and discrimination processes^19^. The N1 component is known to have separable anterior and posterior subcomponents, with the anterior N1 (N1a) peaking earlier over fronto-central electrodes, and the posterior N1 peaking later over lateral posterior occipital electrodes^19,20^. The N1a has been suggested to reflect the top-down (i.e., voluntary, endogenous) control of spatial attention^20,21^, controlled by the dorsal frontoparietal network^22^, while the posterior N1 has been linked to exogenous (i.e., bottom-up) object-based attentional selection in the ventral network^20,21^.

## Neural signatures of perceptual expectations

Neural signatures of perceptual expectations are thought to be dissociable from attentional effects^1,23^. In classical oddball paradigms, for example, responses to unpredictable (*deviant*) stimuli are compared with responses to repeatedly presented stimuli that form a predictable sequence. Predictable (*standard*) stimuli elicit smaller responses than unpredictable (*deviant*) stimuli. Consistent with hierarchical predictive coding models of perception^24,25^, reduced responses to predicted stimuli may be interpreted as reduced prediction errors. Prediction errors are defined as feedforward signals resulting from a comparison of sensory input with top-down predictions generated by higher cortical levels. Prediction errors encode portions of the sensory input that is yet unaccounted for by predictive signals, thereby enabling the formation of accurate percepts by updating an internal generative model of the environment. This results in an increased prediction error signal for unexpected stimuli and a reduced prediction error in response to stimuli that are predicted by the model based on their prior probability. Thus, an attenuation of ERPs as a function of expectations may be interpreted as reduced prediction errors^26,27^. As follows, amplitude suppressions of the N1 component in response to self-induced or self-generated auditory^1^ or visual stimuli^28,29^, temporally predictable stimuli^30,31^, and repeated stimuli^32^, have been interpreted as reduced prediction errors as a function of expectations.

## Attentional gain is influenced by expectations

The degree of endogenous attentional engagement has been shown to be influenced by different kinds of probabilistic manipulations. Different trial histories resulting in varying proportion of validly cued relative to invalidly cued trials^33–36^, different perceptual-motor expectancies^37^, and statistical regularities in sequences^38^, all seem to modulate the size of attentional effects.

Attentional effects are also dependent on task-assignment. For instance, ERP effects observed in probabilistic spatial cueing tasks seem to differ depending on whether attention is engaged on one location in a sustained fashion or allocated transiently on a trial-by-trial basis, as well as whether a behavioural response is required to attended stimuli only or to both attended and unattended stimuli^30,39^. It has been suggested that different results between studies may be attributed to two potentially interwoven mechanisms, those of attentional gain and perceptual expectations^2,30,40^. When attention is manipulated probabilistically, an increase in stimulus probability may generate perceptual expectations^30^.

## Attention and perceptual expectations interact to optimise perception

In the predictive coding framework, attention is understood as a gain mechanism that modulates the variability or precision of prediction errors^41^. The neuronal gain of ascending prediction errors is modulated by expectations about their variability or precision^42^. An interactive pattern between attention and expectations is hypothesized, as predictability leads to an increased precision and, therefore, the attentional gain is increased for expected vs. unexpected sensory input^43^. In studies that have manipulated attention and expectations independently, interactive effects of attention and expectation have indeed been observed. In an fMRI study with a modified version of a cueing task^44^, stimuli at expected spatial locations elicited an attenuated BOLD response in the primary visual cortex (V1) relative to stimuli at unexpected locations when they were unattended (i.e., task-irrelevant). However, a reversed pattern was observed in the attended (i.e., task-relevant) condition, showing an increased BOLD to stimuli at expected relative to unexpected spatial locations. Furthermore, an fMRI study using multi-voxel pattern analysis (MVPA) have reported that attention increases the disparity between representations of expected vs. unexpected stimuli in category-specific visual areas^45^. Similarly, an auditory ERP study^46^ showed that the amplitude of the N1 component was highest in response to tones that appeared in the attended and predictable stream of stimuli relative to attended/unpredictable, unattended/predictable, and unattended/unpredictable conditions. These observations seem consistent with the precision-weighting hypothesis. However, several other recent EEG studies that investigated the potential interrelation between attention and expectations have revealed different patterns of results^47,48^. In our recent study^48^, we used the modified spatial cueing task proposed by Kok *et al*.^44^ and identified distinct stages of interactive influence of attention and prediction on visual ERPs. We observed independent effects of attention and prediction on the amplitude of the posterior-occipital N1 component, corroborating the hypothesised attentional gain enhancement by attention, and the attenuation by prediction. An interaction between attention and prediction was observed within 200 ms, albeit reflected in the selective modulation by expectations in the unattended condition, presumably in the higher-level areas of the dorsal attention network. This interaction effect also showed larger attentional modulation of predicted compared to unpredicted stimuli.

## The present study: rationale and a priori hypotheses

There are several important differences between studies on the interactive influences of attention and prediction that may contribute to discrepant patterns of findings. First, in some studies expectations have been manipulated by instruction or cues^44,48^, while, in other studies, predictability was manipulated in an implicit manner^46^. It has been shown that, if both attention and prediction are manipulated by instructions or cues, task-relevance and probabilistic information are integrated in the pre-stimulus period^48^, which complicates teasing apart their effects on sensory responses evoked by a forthcoming stimulus. Second, in some studies, the attention and expectation manipulations concerned a common feature (e.g., spatial location^44,48^, or timing^49^). In such a situation, full orthogonality may not be present as information provided by predictive cues is inherently task-relevant and therefore requires attentional processing. To gain a deeper understanding on these interactive effects, it seems crucial to probe whether attention and prediction interactively influence ERP responses even if they are manipulated in a fully orthogonal manner.

In the current study, we used a novel variant of a spatial cueing paradigm to achieve a fully orthogonal manipulation of spatial attention and probabilistic feature expectations. Spatial attention was manipulated in a sustained fashion by cues that instructed to attend to grating stimuli appearing in one visual field throughout an experimental block. Expectations were manipulated in a probabilistic fashion and were related to an independent feature of the gratings, namely their orientation. Within one block, gratings of one orientation were presented with higher probability compared to gratings of the other orientation. Importantly, both attention and prediction were manipulated in a sustained manner. Feature expectations were task-irrelevant to ensure that the manipulation of attention and expectations would be fully orthogonal.

We expected a general increase in P1 and N1 amplitudes due to attentional selection. Moreover, we hypothesised that these signatures of attentional gain would be influenced by perceptual expectations and, therefore, we expected to observe an interactive pattern between attention and prediction. Based on previous studies demonstrating that the N1 component is sensitive to both attentional selection and prediction^46,50^, we assumed that an interaction between attention and prediction would be observed in the time window of the N1 component, with the largest N1 amplitude for predictable and attended stimuli. Based on our previous study with a similar task parameters^48^, we expected the stimuli to evoke strongly lateralized posterior-occipital responses. Therefore, we also explored asymmetries of visual evoked potentials at parieto-occipital sites by subtracting ipsilateral from contralateral activity^51^, and we assessed effects of attention and perceptual expectations on event-related lateralisations (ERL), which are considered markers of selective attention: P1pc, N1pc, N2pc. We assumed that attention and perceptual expectations may modulate the N1pc, i.e., the lateralised analogue to the posterior-occipital N1.

## Methods

### Participants

Using G*Power 3.1^52^ software and referring to the observed effect size of the prediction effect (i.e., the comparison of the predicted vs. unpredicted condition) in the time window of the N1 component in our previous study^48^, a sample of 17 participants was estimated to achieve power of ~0.85 (with the significance level set at *p* = 0.05). We recruited twenty-four volunteers through a database of participants at the University of Leipzig. One participant was excluded from further analysis due to a technical failure of EEG recording, two because they did not maintain eye fixation (detected based on eye-tracking data; see below), and three due to excessive motor artefacts in the EEG signal. The remaining 18 participants (13 female, 5 male) with a mean age of 24 years (*SD* = 4, range: 19–30) were predominantly right-handed (lateralisation quotient^53^: M = 89%, SD = 22%, range = 17%–100%), had normal or corrected-to-normal vision, and no history of psychiatric or neurological impairment. After being informed about the nature of the study, they gave written informed consent to participate. They either received course credits or were reimbursed for their participation (€ 6 per hour). The ethics approval for the study was obtained from the Ethics Committee of the Faculty of Medicine at the University of Leipzig, and the study was conducted in accordance with the approved guidelines and regulations.

### Stimuli and apparatus

The stimulus display consisted of a Gabor patch (4.8° × 4.8° sinusoidal grating enveloped by a Gaussian, *SD* = 0.69°) embedded in random noise smoothed with a Gaussian filter (*SD* = 0.69°), and centred on the horizontal meridian 3.5° to the left and right of the fixation cross. The orientation of the Gabor patch was either 45° or 135°, with spatial frequency of either 2.6 cycles per degree (cpd) or 1.7 cpd. The phase was pseudorandomised and sampled from the range of 0° to 330° in 16 steps of 22°. The stimulus was presented on a grey background. The stimulus contrast was adjusted individually for each participant in a weighted up-down adaptive procedure (see below). Stimuli were created, presented, and responses to them collected using MATLAB (The Mathworks, Inc, Natick, MA) in conjunction with Psychophysics Toolbox 3^54,55^. The experimental procedure was presented on a 19” CRT monitor (G90fB, ViewSonic, Walnut, CA; resolution 1024 × 768 pixels, refresh rate of 100 Hz). Participants viewed the display from a distance of 57 cm with their heads on a chinrest. The experiment was conducted in a dimly lit and electrically shielded chamber.

### Procedure

**Figure 1:**
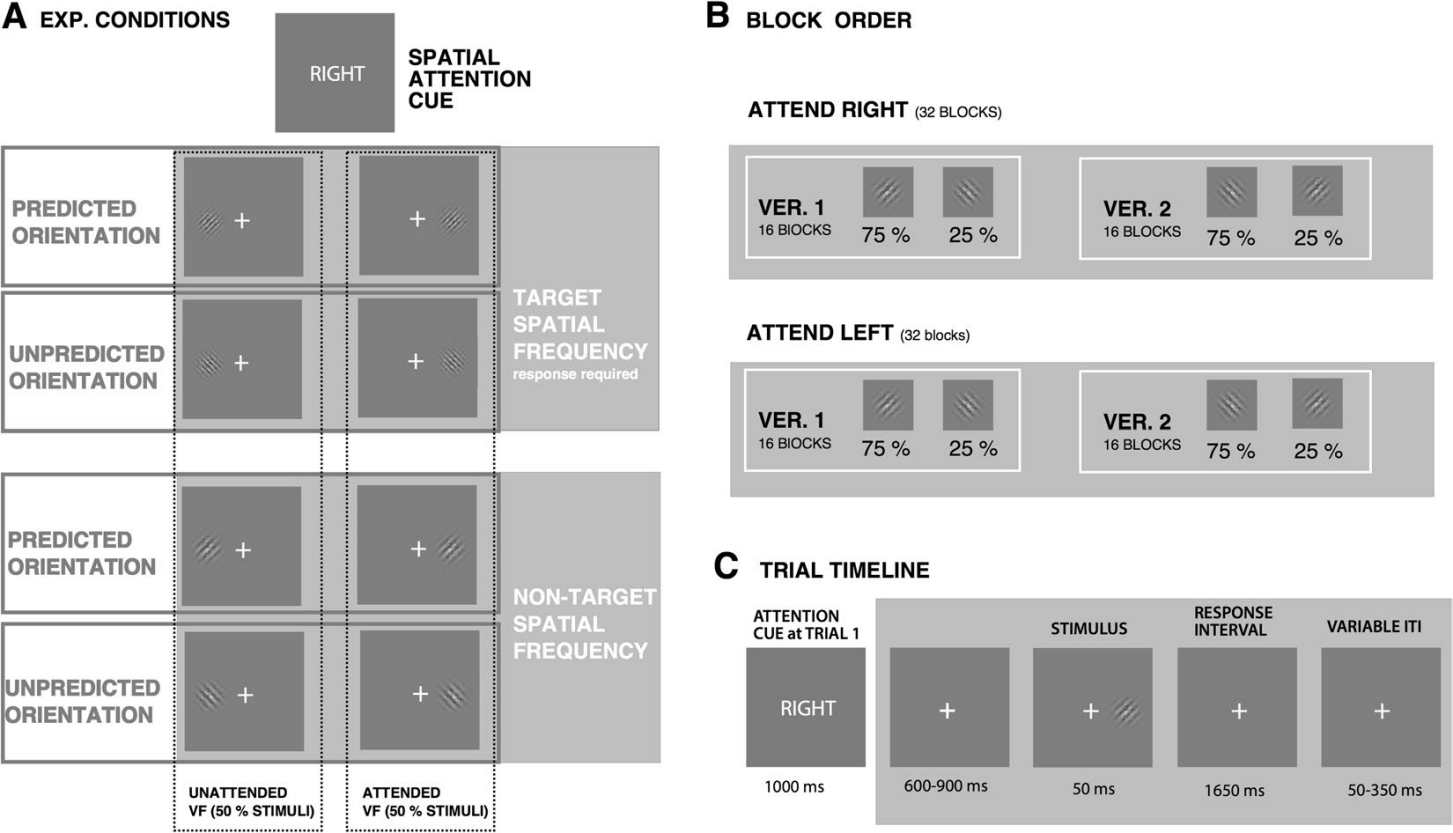
Experimental procedure (**A)** Stimulus conditions (note that response requirements with respect to spatial frequency were counterbalanced between participants). (**B)** Block order (note that the order of both attention cueing conditions and orientation-probability contingency was counterbalanced across participants). (**C)** Trial timeline.

Each block started with a presentation of an attention cue (words ‘LEFT’ or ‘RIGHT’), which instructed participants to attend to the left visual field (LVF) or the right visual field (RVF) throughout the block (see Fig. 1A). Attention cues were presented in the centre of the screen for 1000 ms (see Fig. 1C). Each trial started with a presentation of a cross (“+” sign) at the centre of the screen that participants were required to fixate throughout the whole experiment. After a variable interval (600–900 ms), a Gabor patch was presented for 50 ms randomly either in the LVF or RVF. Perceptual expectations were manipulated probabilistically by presenting Gabor patches of more likely (predicted: 75% of Gabor patches within block) or a less likely (unpredicted: 25% Gabor patches) orientation. Within block version 1, 75% of Gabor patches had 45° orientation while 25% of Gabor patches had 135° orientation, and vice-versa for block version 2 (see Fig. 1B). Gabor patches were of high (i.e., 2.6 cpd; 50% of stimuli) or low (i.e., 1.7 cpd; 50% of stimuli) spatial frequency. Participants were asked to perform a discrimination task only on the attended side and to respond to either high or low spatial frequency by pressing a designated button on a response box with their right hand. The response instruction was counterbalanced between participants, so that half of the participants responded to higher spatial frequency gratings and another half responded to lower spatial frequency gratings. The response window was 1650 ms, followed by an inter-trial interval varying between 50–350 ms (see Fig. 1C).

The blocks consisting of 16 trials were administered in a fixed order: 32 blocks of the task with the same cued VF (e.g., ‘LEFT’ attention cue) were followed by 32 blocks in which the other VF was cued (e.g., ‘RIGHT’ attention cue). Within 32 blocks with the same attention cue, 16 blocks of the task had one orientation-probability contingency (i.e., block version 1; see Fig. 1B) and were followed by 16 blocks of another orientation-probability contingency (i.e., block version 2; see Fig. 1B). The order of both attention cueing conditions and orientation-probability contingency was counterbalanced across participants. Experimental blocks were preceded by a training run in which participants were familiarised with 4 blocks of the task (one block of each kind). The task consisted of 1024 trials in total, divided in 64 blocks. After each 4 blocks, participants could rest for a variable period of time.

The stimulus contrast was adjusted at the beginning of the experiment, using an adaptive staircase procedure^56^. In this procedure, the trial sequence and timing were kept identical to the experimental task, but attention cues were not included, and participants were asked to identify the spatial frequency of patches appearing in both visual fields. The just noticeable difference was set at 90%. The task was repeated at least twice and the contrast value calculated from the last run was used in the experiment (*M* = 0.18, *SD* = 0.04).

### Eye tracking

To ensure that participants maintained fixation during the trials, we recorded their eye movements with an infrared eye-tracking system (EyeLink 1000; SR Research Ltd., Mississauga, Ontario, Canada) at a sampling rate of 500 Hz. The monocular recordings were controlled by the EyeLink Software. Due to observed horizontal and vertical offset of eye fixation, eye-tracking data were corrected by subtracting a median offset calculated for each block. Subsequently, trials during which gaze was not fixated on the area 1° degree around the fixation cross at the time of the presentation of the grating (50 ms) were excluded from the EEG analysis (*M* = 5.2% per condition and participant, range: 0–20.3%). If participants did not maintain central fixation in more than 50% of the trials, their data were not included in analyses (2 participants, see above).

### EEG recording

The electroencephalogram (EEG) was continuously recorded at a sampling rate of 500 Hz from 59 Ag/AgCl active electrodes using a BrainAmp amplifier and the Vision Recorder software (Brain Products™ GmbH, Munich, Germany). Electrodes were mounted into an elastic cap (actiCAP) following the extended international 10–20 system^57^. An electrode placed on the tip of the nose served as an online reference, a ground electrode was placed on the forehead, and two electrodes were attached to the earlobes for offline re-referencing. Electrooculogram (EOG) was recorded using electrodes placed at the outer canthi and below and above (electrode Fp1) the left eye.

### EEG data preprocessing

EEG preprocessing was carried out using EEGLAB^58^. Data were re-referenced offline to the average of the left and right earlobes. Vertical electrooculogram (VEOG) and horizontal electrooculogram (HEOG) were calculated from the EOG data. The data were filtered with a 0.1 Hz high-pass and a 40 Hz low-pass windowed sinc finite impulse response (FIR) filter (Hamming window, filter order 8250 and 184 for high-pass and low-pass filter, respectively). Independent Component Analysis (ICA) was used to remove eye-blinks, muscle artefacts, and noisy channels from continuous data, based on measures computed with FASTER (correlation with EOG channels, spatial kurtosis, power spectrum slope, Hurst exponent)^59^ and SASICA (low autocorrelation, focal topography, correlation with HEOG and VEOG)^60^. On average, 3.4 components per participant were removed (range: 1–6). Noisy channels whose data’s joint probabilities exceeded a threshold of 3 standard deviations were excluded and interpolated using spherical interpolation (on average 1.8 channels, range: 0–4). Subsequently, stimulus-locked epochs of −200 to 500 ms were defined, and baseline corrected using the 200 ms window before stimulus presentation. Finally, epochs with an amplitude change exceeding 75 µV on any channel were rejected from further analysis.

### Behavioural and EEG data analyses

The effectiveness of attention manipulation was assessed in the behavioural data by calculating the proportion of false alarms (FA) in the unattended condition (i.e., responses to stimuli that appeared on the unattended side). To assess the accuracy in the spatial frequency discrimination task, the proportion of false alarms for attended Gabor patches of spatial frequency that did not require a response and proportion of misses for Gabor patches of response-relevant spatial frequency were calculated. The effects of the probabilistic manipulation were assessed by comparing, using paired-sample *t*-tests, mean response times to predicted vs. unpredicted stimuli (i.e., more likely or less likely appearing in the block).

Discrimination performance was analysed based on signal detection theory (SDT)^61–63^. The proportion of trials in which stimuli with a task-relevant spatial frequency at the attended side were correctly identified were considered as *hits*. Trials, in which stimuli with task relevant spatial frequency were presented at the attended side, but were not responded to, were defined as *misses*. Trials, in which stimuli with task-irrelevant spatial frequency were presented at the attended side and they were responded to, were defined as *false alarms*. Trials in which stimuli with task-irrelevant spatial frequency were presented at the attended side and response to them was correctly withheld, were defined as *correct rejections* ^64–66^. Based on the proportions of hits, misses, false alarms and correct rejections, non-parametric estimates of sensitivity (*A’*) and response bias (*B”*_*D*_) were calculated. *A’* ranges from 0.5 (signal is indistinguishable from noise) to 1 (perfect performance)^67^. *B”*_*D*_ ranges from −1 to 1, these values signifying extreme bias in favour of *no* (i.e., reporting an absence of task-relevant spatial frequency) vs. *yes* (i.e., a reporting a presence of task-relevant spatial frequency) responses^68,69^, whereas 0 indicates no response bias. *A’* was compared against 0.5 (chance level) and *B”*_*D*_ was compared against 0 (no response bias) by means of one-sample *t*-tests. These two measures were also compared between unpredicted and predicted conditions using paired-sample *t*-tests.

Average ERP waveforms were computed separately for each participant and condition, for channels contralateral (i.e., left hemisphere for RVF stimuli and right hemisphere channels for LVF stimuli) and ipsilateral (i.e., right hemisphere channels for RVF stimuli and left hemisphere channels for LVF stimuli) to the side at which the stimulus was presented. For the predicted condition, we included only trials, which directly preceded unpredicted trials, in order to balance the number of trials across conditions. In the unpredicted condition, we excluded trials that were repetitions of unpredicted orientation, in order to avoid potential confounding effects of stimulus repetition. The resulting mean amount of trials per condition and participant was 79 (SD = 15, range: 44–103; attended/predicted: M = 79, SD = 18; attended/unpredicted: M = 79, SD = 14, unattended/predicted: M = 79, SD = 10, unattended/unpredicted; M = 78; SD = 16). A grand mean was calculated by averaging each condition across participants.

The N1 component, identified via visual inspection, was characterised by an anterior distribution; therefore, mean amplitudes in the cluster of six fronto-central electrodes (‘F1/2i’, ‘Fz’, ‘F1/2c’, ‘FC1/2i’, ‘FCz’, ‘FC1/2c’) in the time window of 150–196 ms (±23 ms around the peak of the component) were analysed. A repeated measures ANOVA (rANOVA) including factors attention (attended, unattended), prediction (predicted, unpredicted), and electrode location (ipsilateral: ‘F1/2i’ and ‘FC1/2i’, midline: ‘Fz’ and ‘FCz’, contralateral: ‘F1/2c’ and ‘FC1/2c’), was conducted.

To capture asymmetries of early visual evoked potentials at parieto-occipital sites, ERLs were computed by subtracting ipsilateral from contralateral activity sites. Three ERLs were assessed in the cluster of three lateral posterior electrodes (PO7/8c-i, P7/8c-i, P5/6c-i), namely the P1pc (76–106 ms; ±15 ms around the peak amplitude), N1pc (136–186 ms; ±25 ms around the peak amplitude), and N2pc (242–288 ms; ±23 ms around the peak amplitude).

We additionally analysed the effects of attention and prediction on the contralateral and ipsilateral P1 components, as well as P3 subcomponents – P3a and P3b (see Supplementary Information).

### Details of the statistical procedures

Statistical analyses were performed in *R 3*.*3*.*1* ^70^, using packages *ez* v*4*.*3* ^71^, *MASS 7*.*3-45* ^72^, *car 2*.*1-2* ^73^, and all the respective dependencies.

The significance level for all frequentist tests was set at *p* = 0.05. In case the assumption of normality was violated – as indicated by statistically significant Shapiro-Wilk tests – Box-Cox transformation was performed^74^ to identify the lambda value with the highest log-likelihood. With respect to rANOVAs, Greenhouse-Geisser corrected *p*-values were reported in case the assumption of sphericity was violated, and generalized eta squared was used as a measure of effect size^75^. Paired comparisons were conducted by means of paired-sample *t*-tests, and Pearson’s *r* was used as a measure of effect size^76^.

Frequentist analyses were complemented by estimating Bayes Factors (*BF*_10_)^77–81^. For the Bayesian rANOVA, participants were included in all models as a random factor and their variance was considered as nuisance. We focused on the subset of all models, in which an interaction can be included only if all constituent effects or interactions are also included (i.e., attention, prediction, and their interaction; or attention, prediction, lateralisation, and their interaction)^81^. We compared these models against the null model (i.e., including only the random factor). In addition, we compared the BF_10_ of the full model (i.e., all main effects and interactions) with models that included only main effects, in order to better characterize the independent contribution of attention and prediction. The calculation of BF_10_ was performed using the *BayesFactor 0*.*9*.*1*2*–*2 package^81,82^ using 10,000 Monte-Carlo sampling iterations. The null hypothesis was specified as a point-null prior (i.e., standardized effect size *δ* = 0), whereas the alternative hypothesis was defined as a Jeffrey-Zellner-Siow (*JZS*) prior, i.e., a folded Cauchy distribution centred around *δ* = 0 with a scaling factor of *r* = 0.707^79,80^. Data were interpreted as positively in favour of the null (or alternative) hypothesis if BF_10_ was at least lower than 0.3 (or larger than 3), whereas BF_10_ close to 1 would be only weakly informative^77,78^.

### Data availability

The data generated and analysed during the current study are available from the Open Science Framework, https://osf.io/rqvh3.

## Results

### Behavioural results

The mean proportion of false alarms in the unattended condition was 0.0004 (*SD* = 0.001), suggesting that participants followed the instruction provided by the attention cues. The mean proportion of false alarms for attended stimuli that did not require a response was 0.16 (*SD* = 0.13) and the mean proportion of missed responses to attended and task-relevant spatial frequency was 0.09 (*SD* = 0.07). The mean response time in the unpredicted condition was 601 ms (SD = 74), while the mean response time in the predicted condition was 600 ms (SD = 69). These means were not statistically different (*M* = 0.33 ms, *t*_17_ = 0.06, *p* = 0.953, *r* = 0.01). Bayesian analysis indicated positive evidence in favour of the null hypothesis, BF_10_ = 0.24 ± 0.01%.

The analysis of signal detection measures showed that discrimination performance (*A’*) was above chance in both the predicted (*M* = 0.92, *SD* = 0.05, *t*_17_ = 38.74, *p* < 0.001, *r* = 0.99) and unpredicted conditions (*M* = 0.93, SD = 0.05, *t*_17_ = 40.63, *p* < 0.001, *r* = 0.99). *A’* was not statistically different between unpredicted and predicted conditions (*M* = 0.002, *t*_17_ = 0.44, *p* = 0.662, *r* = 0.11). Bayesian one-sample *t*-test showed very strong evidence for above-chance performance in both the predicted (BF_10_ = 6.11 × 10^14^ ± 0%) and unpredicted conditions (BF_10_ = 1.29 × 10^15^ ± 0%). Bayesian paired *t*-test comparing the two conditions indicated positive evidence in favour of the null hypothesis, BF_10_ = 0.26 ± 0%. Analysis of response bias (*B”*_*D*_) revealed a significant bias towards *yes* (i.e., a bias towards reporting a presence of task-relevant spatial frequency) responses in the predicted condition, *M* = −0.36, *SD* = 0.50, *t*_17_ = −3.08, *p* = 0.007, *r* = 0.60, while the bias did not seem to be statistically reliable in the unpredicted condition, *M* = −0.27, *SD* = 0.62, *t*_17_ = −1.84, *p* = 0.083, *r* = 0.41. The difference between the unpredicted and the predicted condition was not significant, *M* = 0.10, *t*_17_ = 1.64, *p* = 0.119, *r = *0.37. Bayesian analysis showed evidence for response bias in the predicted condition, BF_10_ = 7.31 ± 0%, while the data were not informative for the unpredicted condition, BF_10_ = 0.98 ± 0%. Bayesian *t*-test comparing the two conditions indicated only anecdotal evidence for the null hypothesis (BF_10_ = 0.75 ± 0%).

### EEG results

#### N1a

**Figure 2:**
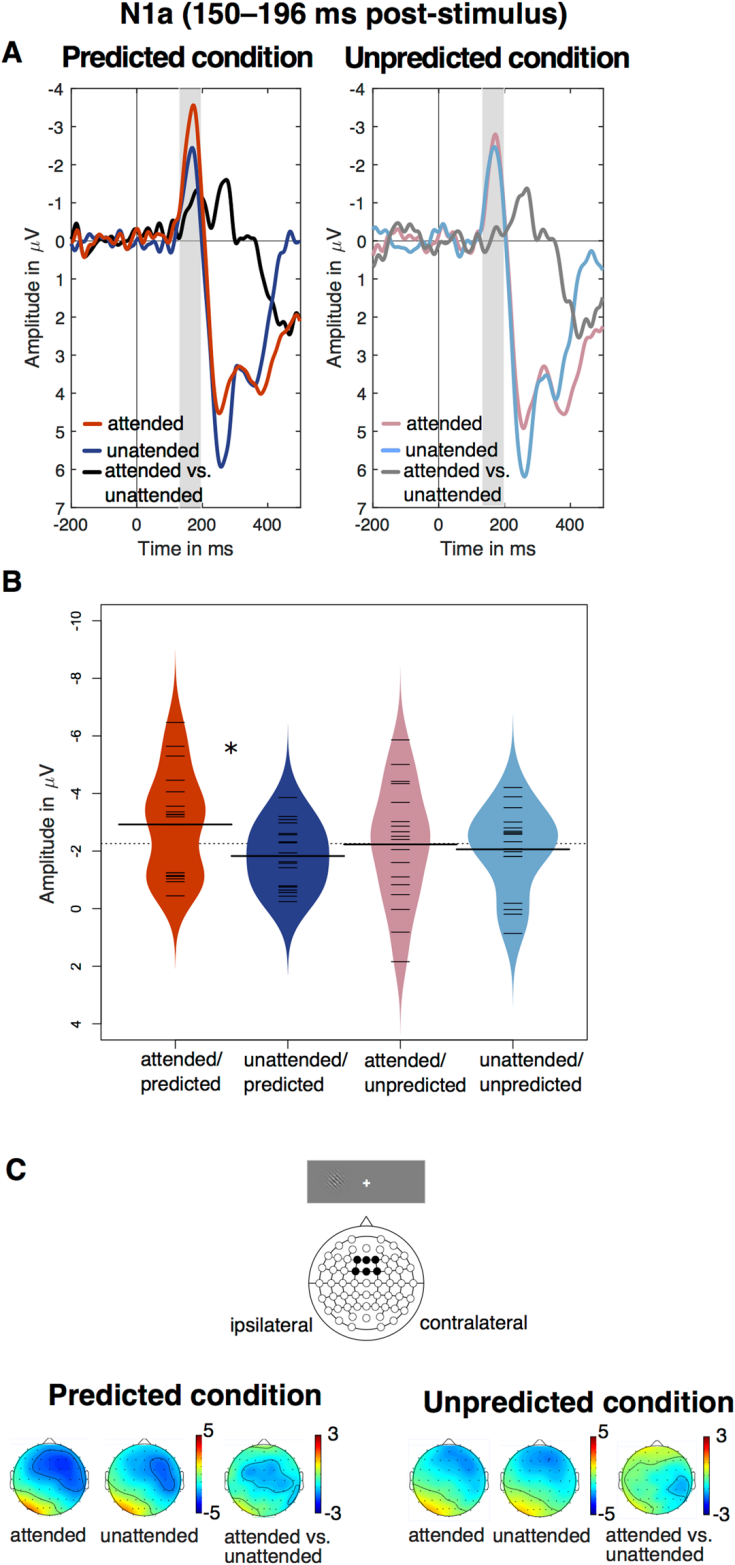
(**A**) ERPs from the averaged cluster of fronto-central electrodes (F1/2i’, ‘Fz’, ‘F1/2c’, ‘FC1/2i’, ‘FCz’, and ‘FC1/2c’), depicting the attentional modulation in the predicted and unpredicted condition respectively. The N1a time window (150–196 ms) is marked with grey panels. (**B**) Beanplots^102^ showing the interaction effect in the N1a time window. Thick horizontal lines represent means; thin lines represent individual data points; and coloured parts represent estimated density of distributions. (**C**) Topography of the attentional modulation of the N1a in the predicted and unpredicted condition.

In the fronto-central electrode-cluster, a three-way rANOVA (*attention*: attended, unattended; *prediction*: predicted, unpredicted; *electrode location*: ipsilateral, midline, contralateral) on N1 amplitude values between 150–196 ms post-stimulus onset revealed a significant main effect of electrode location (*F*(2,34) = 10.90, *p* < 0.001, *η* ^2^_*G*_ = 0.010), with more negative amplitudes at the contralateral (*M* = −2.47 µV, *SD* = 1.26 µV) than midline (*M* = −2.24 µV, *SD* = 1.24 µV) or ipsilateral electrodes (*M* = −2.07 µV, *SD* = 1.12 µV; see Fig. 2). Furthermore, a significant interaction between attention and prediction was observed (*F*(1,17) = 6.60, *p* = 0.020, *η* ^2^_*G*_ = 0.020), indicating that the N1 was most negative for attended and predicted stimuli (see Fig. 2). In the predicted condition, a significant difference between attended and unattended stimuli was found (*M* = −1.10 µV, *t*_17_ = −2.58, *p* = 0.019, *r* = 0.53), while the responses to attended and unattended stimuli did not significantly differ in the unpredicted condition (*M* = −0.17 µV, *t*_17_ = −0.37, *p* = 0.717, *r* = 0.09). The main effects of prediction and attention were not significant, *F*s < 2.41, *p*s > 0.14, *η* ^2^_*G*_ < 0.037, neither was a three-way interaction between attention, prediction, and electrode location, *F*(2,34) = 0.80, *p* = 0.458, *η* ^2^_*G*_ = 0.001.

Bayesian analysis showed positive evidence for the null model compared to the full model (BF_10_ = 0.01 ± 12.57%). However, strong evidence in favour of a model that included a main effect of attention, a main effect of prediction, and their interaction, was observed, BF_10_ = 173.87 ± 5.84% (see Table 1). Bayesian *t*-tests were used to compare the magnitude of the attention effect separately in the predicted and the unpredicted condition. In the predicted condition, the alternative hypothesis (i.e., attention effect) was 3.08 times more likely than the null hypothesis (BF_10_ = 3.08 ± 0%). In the unpredicted condition, evidence in favour of the null hypothesis (i.e., no attention effect) was observed (BF_10_ = 0.26 ± 0.01%). The finding of a reliable attention effect only in the predicted condition, but not in the unpredicted condition, conforms to the hypothesis that attentional gain and probabilistic feature expectations modulate the N1a component in an *interactive* manner.

**Table 1:** Bayes factors (BF_10_) and percentage of proportional errors (% pe) for each model of interest, obtained by using JZS priors with a scaling factor of *r* = 0.707 (see Methods and Results sections for details). The models with the best explanatory power are highlighted in bold.

Component	Model	BF_10_	± % pe
**anterior N1** (150–196 ms)	Att	100.64	1.08
	Pred	0.25	1.47
	Lat	0.14	0.76
	Att + Pred	25.78	1.32
	Att + Pred + Lat	4.61	3.75
	**Att** **+** **Pred** **+** Att*Pred	**173.87**	**5.84**
	Att + Pred + Lat + Att*Pred + Lat + Pred*Lat + Rel*Lat + Pred*Rel*Lat	0.01	12.57
**P1pc** (76–106 ms)	Att	0.18	1.19
	Pred	0.49	1.31
	Att + Pred	0.09	6.64
	Att + Pred + Att*Pred	0.35	3.01
**N1pc** (136–186 ms)	**Att**	**63,554.46**	**0.83**
	Pred	0.34	1.16
	Att + Pred	33,230.40	2.07
	Att + Pred + Att*Pred	8,544.00	3.08
**N2pc** (244–288 ms)	Att	**25.02**	**0.92**
	Pred	0.189	1.02
	Att + Pred	4.72	1.34
	Att + Pred + Att*Pred	1.33	4.52

#### ERLs: P1pc, N1pc, N2pc

A two-way rANOVA on the P1pc (76–106 ms after stimulus onset) showed that this component did not seem to be reliably modulated by attention (*F*(1,17) = 0.01, p = 0.916, *η* ^2^_*G*_ < 0.001) or prediction (*F*(1,17) = 4.15, *p* = 0.057, *η* ^2^_*G*_ = 0.019). An interaction between prediction and attention also exceeded the significance level, *F*(1,17) = 4.29, *p* = 0.054, *η* ^2^_*G*_ = 0.043. Bayesian analysis showed anecdotal evidence against the full model when compared to the null model, BF_10_ = 0.35 ± 3.02%. This was also the case for the model with the highest BF_10_, namely a model including prediction (BF_10_ = 0.49 ± 1.31%; see Table 1). Therefore, no reliable modulation of the P1pc by attention or prediction has been observed conclusively.

**Figure 3:**
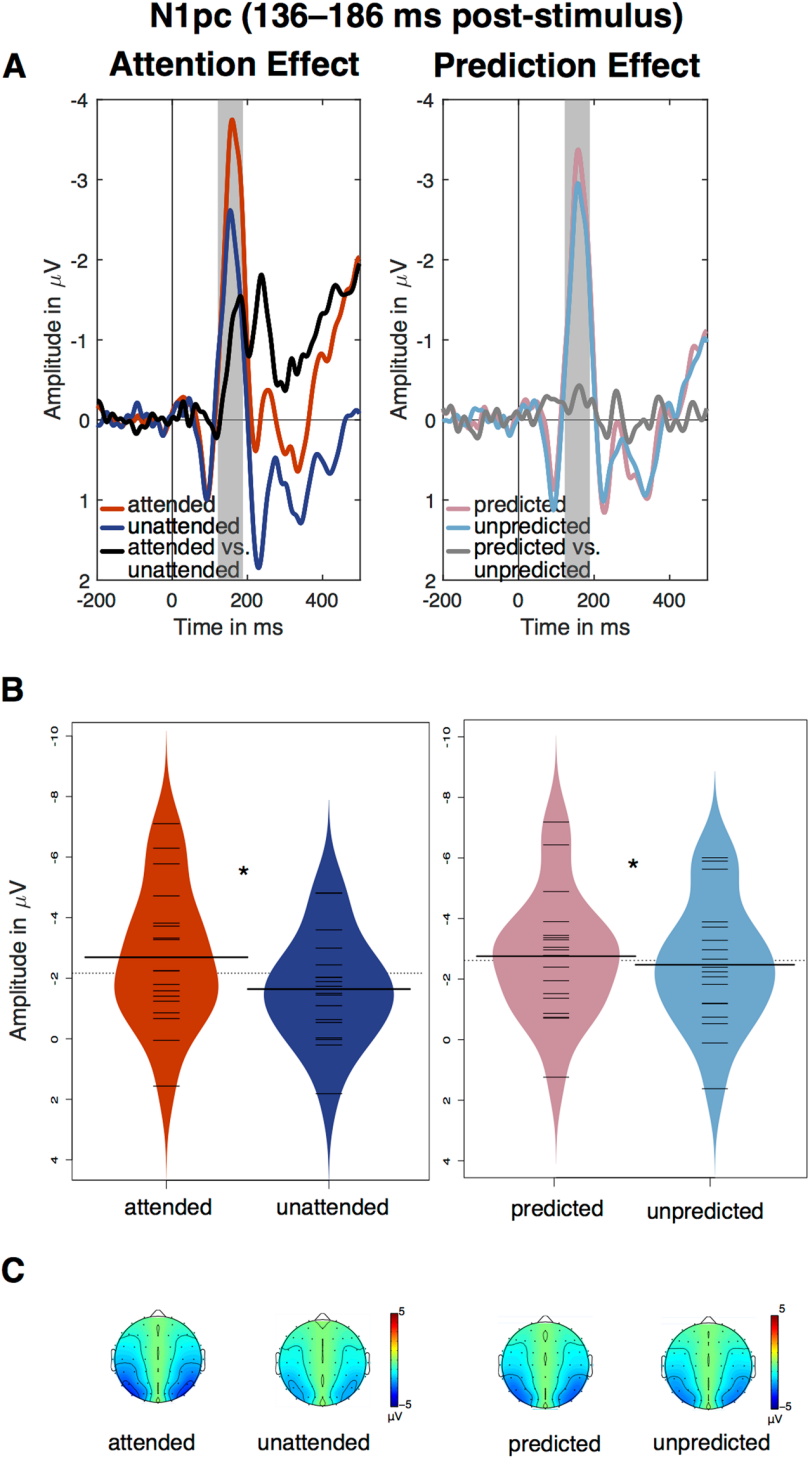
(**A**) ERLs of the main effects of attention and expectations in the averaged cluster of lateral posterior electrodes (PO7/8c-i, P7/8c-i, P5/6c-i). The N1pc time window (136–186 ms) is marked with grey panels. (**B**) Beanplots depicting the effects of attention and prediction in the N1pc time window. (**C**) Topographies of the N1pc in the respective conditions. The same topography (contralateral-ipsilateral ERP) is plotted in the left and the right hemisphere.

In the time window of the N1pc component (136–186 ms post-stimulus onset), a significant main effect of attention was observed, *F*(1,17) = 18.47, *p* < 0.001, *η* ^2^_*G*_ = 0.058. Attended stimuli elicited a larger lateralised negativity (*M* = −3.17 µV, *SD* = 2.39) than unattended stimuli (*M* = −2.06 µV, *SD* = 1.80; see Fig. 3). The N1pc was also larger for predicted (*M* = −2.76 µV, *SD* = 2.06) relative to unpredicted stimuli (*M* = −2.47 µV, *SD* = 2.07; see Fig. 3), as evidenced by a significant main effect of prediction (*F*(1,17) = 4.69, *p* = 0.045, *η* ^2^_*G*_ = 0.004). The interaction between attention and prediction was not significant, *F* = 0.16, *p* = 0.690, *η* ^*2*^_*G*_ < 0.001. Bayesian analysis indicated that the model including the main effect of attention (BF_10_ = 63,554.46 ± 0.83%) was 7.44 more likely than the full model. The model including both main effects, attention and prediction, was 3.89 times more likely than the full model. Moreover, the model including main effects of attention and prediction (BF_10_ = 33,230.40 ± 2.07%) was found to be only 0.52 less likely than the model including attention only; however, the BF value does not allow to reliably adjudicate which of these models should be treated as preferred. Taken together, these results show that the lateralized N1pc component has been modulated by attention and probabilistic expectations in an *additive* manner.

In the time window of the N2pc component (244–288 ms post-stimulus onset), amplitude was more negative in the attended (*M* = −0.16 µV, *SD* = 2.36) than the unattended condition (*M* = −0.78 µV, *SD* = 2.11), as evidenced by a significant main effect of attention (*F*(1,17) = 5.01, *p* = 0.039, *η* ^*2*^_*G*_ = 0.040). The main effect of prediction and the attention × prediction interaction were not significant, *Fs* < 0.67, *p*s > 0.501, *η* ^*2*^_*G*_ < 0.001. Bayesian analysis indicated that the model with a main effect of attention should be preferred over the null model (BF_10_ = 25.02 ± 0.92%). In addition, this model was 18.81 times more likely than the full model, BF_10_ = 1.33 ± 4.52% (see Table 1). The result shows that the N2pc, a marker of selective attention, is higher for attended vs. unattended stimuli, but it is not modulated by perceptual expectations.

## Discussion

The current study investigated whether early electrophysiological signatures of attentional gain are modulated by probabilistic feature expectations. We used a novel version of the spatial cueing task, in which cues instructed participants to attend to a given visual field throughout each block and to discriminate gratings based on their spatial frequency. Conditional probability of the gratings’ orientation was manipulated within blocks, so that the orientation was either predicted (more likely) or unpredicted (less likely). Thus, spatial attention and perceptual expectations were manipulated orthogonally. We analysed the signature of attentional gain reflected by the modulation of the N1 component. We hypothesised that attentional gain would be modulated by prediction about features of visual stimuli. If attentional selection is facilitated by stimulus predictability, an interaction between attention and prediction in the time window of the N1 component would be observed.

### Expectations about task-irrelevant features do not influence decision sensitivity, but may influence decision bias

We did not observe clear-cut behavioural effects of perceptual expectations. Previous studies have shown that context-specific expectations may influence decision sensitivity^83,84^. However, according to a framework proposed by Summerfield and Egner^5^, feature expectations generated on the basis of prior probability, rather than the decision sensitivity, are expected to influence a decision criterion. Therefore, they may lead to a response bias, and seem to improve metacognitive judgements^3,9^. It has been suggested that, in experiments studying effects of perceptual expectations, modulations of decision sensitivity may rather be attributed to attentional confounds^5^. In the current study, in which spatial attention and feature expectations were manipulated fully orthogonally, we observed evidence indicating that predicted and unpredicted stimuli did not differ with respect to decision sensitivity. The data, however, were less informative concerning the hypothesis that predictions would modulate response bias. Reliable evidence of response bias was observed in the predicted condition, whereas the data were non-informative concerning the presence of response bias in the unpredicted condition. Statistically ambiguous evidence regarding response bias in the current experiment may be attributed either to low statistical power or methodological differences between previous studies and the present one. Firstly, unlike previous studies^4,9^, expectations were manipulated in an implicit fashion; hence no cue would inform participants about the likelihood of the stimuli. Secondly, our manipulations of expectations were orthogonal with regards to the behavioural task. Previous studies have suggested that predictions related to task-irrelevant dimensions of stimuli may facilitate behaviour^38^ and influence ERP responses reflecting attentional gain^46^. However, at present it is not clear whether explicit top-down information is necessary to observe a clear behavioural influence of expectations.

### Top-down attentional gain is modulated by expectations about stimulus features

To investigate whether attention and perceptual expectations jointly influence early signatures of attentional gain, we focused primarily on the N1 component. We observed that the N1a component has been jointly modulated by attention and perceptual expectations. As hypothesised, the N1a amplitude was largest in response to attended and predicted stimuli. Only for gratings with predicted orientation, the N1a response was larger in the attended relative to unattended condition. On the other hand, no reliable attentional modulation was observed for stimuli with the unpredicted orientation.

The N1 attention effect is commonly associated with attentional facilitation^12,17^. It has been suggested that anterior and posterior-occipital subcomponents of the N1 may reflect different functional roles and have different neural generators. The N1a generators have been localised in the parietal lobe, near the intraparietal sulcus, while sources of the posterior subcomponent have been localised in the extrastriate cortex^85,86^. The modulation of the anterior subcomponent of the N1 may be linked to voluntary (i.e., top-down, endogenous) control of spatial attention in the dorsal frontoparietal network^22^, while the posterior subcomponent of the N1 presumably originates from regions in extrastriate cortex, including the occipital gyrus and ventral fusiform gyrus. Previous studies have suggested that modulations of the posterior N1 may be linked to exogenous (i.e., bottom-up) object-based attentional selection subserved by the ventral network^20,21^. Considering the dissociation between N1 subcomponents, a joint effect of attention and prediction on the anterior N1 component may be linked to a modulation within the dorsal and fronto-parietal network in control of voluntary attention shifts. The current data suggest that attentional facilitation, as indexed by the N1a component, may be contingent on the perceptual expectations about stimulus features.

### ERLs are reliably modulated by attention

We further explored effects of attention and perceptual expectations on posterior occipital components by subtracting ipsilateral from contralateral activity^51^, a procedure that exploits the well-known attention-dependent asymmetrical representation of perceptual environments in the visual system^87^. Among the different ERL components, the N1pc is commonly elicited following unilateral presentation and is assumed to reflect saliency-based, bottom-up attentional orienting^88,89^, followed by the N2pc component thought to index attentional capture by relevant stimuli^90,91^. In the present study, we observed reliable modulations of the N1pc and the N2pc by spatial orienting, consistent with the idea that these components reflect perceptual and attentional tuning to task-relevant features.

### Additive influence of attention and perceptual expectations on the N1pc

A novel finding regarding prediction effects was observed on the amplitude of the N1pc: the N1pc was significantly larger for predicted than unpredicted stimuli. The increase in the N1pc as a function of valid feature expectations runs counter to the hypothesis that responses to expected stimuli would elicit smaller prediction error signals than unexpected stimuli. It could be speculated that perceptual processing may be facilitated by valid probabilistic expectations due to an increase in the precision of prediction errors in stimulus-specific populations (i.e., in the hemisphere contralateral to stimulus). This ‘sharpening hypothesis’ has been corroborated by recent fMRI^92^, ERP^93,94^ and magnetoencephalography (MEG) studies^95^. In particular, Barascud *et al*.^95^ have proposed that the complexity of the predicted stimulus may determine whether responses to predicted stimuli are increased or decreased  compared to responses to unpredicted stimuli. Less complex predicted signals may lead to smaller response amplitudes due to adaptation effects based on low-level transitional probabilities, while complex predicted signals (such as the random noise-filtered grating stimuli with randomised phase used in the present study) may lead to increases in response amplitude due to precision-weighting.

Of note, Bayesian analysis comparing the model that included both main effects (i.e., attention and prediction) with the attention effect only showed very weak evidence in favour of the latter model. The current results do not allow to reliably adjudicate between the two models, therefore, this finding should be treated with caution. Future high-powered studies could arbitrate between the model that considers attentional influence only and a model which considers an additive influence of attention and perceptual expectations on N1pc amplitudes.

### Additive and interactive effects of attention and perceptual expectations

We conclude that perceptual expectations may have differentially influenced dissociable processes assumed to be related to N1 subcomponents. On the one hand, exogenous (‘bottom-up’) attentional capture by task-relevant features was reliably observed independently of expectations about stimulus. Expectations, however, have *additively* influenced the N1pc component, and led to a facilitation of perceptual processing for expected stimuli independent of attention. On the other hand, the voluntary endogenous attention effect, assumed to be reflected in the N1a, was observed for predicted stimuli only and not for the unpredicted stimuli, pointing to an *interactive* influence of attention and expectations, and a possible dependence of top-down attentional facilitation effects on the availability of perceptual expectations about stimulus features.

The observation of interactive effects is consistent with several previous studies. An increased response to predicted and attended stimuli in the V1 was first found in an fMRI study^44^ that employed a modified spatial cueing task, in which attention and prediction were manipulated by two independent cues. Moreover, another study^45^ reported that attention increases disparity between representations of expected vs. unexpected stimuli in category-specific visual areas. A similar pattern of results was reported in an auditory EEG study^46^. Participants were asked to attend to one of two streams of predictable or unpredictable auditory stimuli and to detect tones of attenuated loudness. The auditory N1 was found to be selectively increased for stimuli embedded in an attended and predictable stimulus stream. The N1 attention effect was present only in the predictable condition, while it was not present in the unpredictable streams. Taken together, these data indicate that attentional selection may be facilitated by statistical regularities in the environment^38^. On the other hand, the finding of *additive* effects on early posterior-occipital responses would seem in line with another recent MVPA study, in which expectations and task-relevance additively improved classification accuracy of grating orientation in the V1^92^.

### How do attention and perceptual expectations interact to optimise perception?

According to the predictive coding theory, attention is associated with a mechanism that modulates precision (i.e., the inverse of variability) of ascending prediction errors^24,41,42,96,97^. Prediction errors that encode the content of sensory input, which is yet unexplained by the internal model of the environment, are believed to be modulated by the inference about the precision of prediction errors. The precision is inferred to be higher for predicted, regularly repeating stimuli, which may increase gain of prediction error signals^38,43,95^. The current data provide support for this hypothesis. However, similarly to our previous study^48^, the interplay of attention and expectation has not reliably influenced the posterior parieto-occipital responses presumably reflecting lower-level processing in the unimodal visual areas, which have shown additive influences of attention and expectations. The interplay between attention and expectations, however, was reflected in modulations of the N1a component, presumably related to processing in the higher-level areas of the dorsal fronto-parietal network.

It should also be noted that some recent fMRI and M/EEG studies reported a different pattern of interaction between attention and expectations, whereby a modulation by expectations was either selectively present or more pronounced in the unattended^47,48,50,98^, or conversely, in the attended condition^99,100^. Differences in how the interaction patterns are manifested may be attributed to diverse manipulations of attention and expectations^1^. The interactions between attention and prediction may unfold differently dependent on the information provided by the manipulation of expectation, which may either be contextual in nature^47,48,50,99^, or can relate to the perceptual features of the stimuli, as in the current study. Moreover, the complexity of stimulus features, which may either afford or exclude low-level neural adaptation based on transitional probabilities, needs to be further considered^95,101^. Furthermore, if attention or expectations are manipulated probabilistically, they may provide varying degree of certainty, or confidence, about upcoming stimuli prior to stimulus presentation, leading to baseline shifts^41^. Future studies could investigate how these issues contribute to the interactive top-down influences on sensory processing.

## Conclusions

To summarise, attention and prediction seem to *interactively* optimise visual perception within 200 ms after stimulus onset. When spatial attention and perceptual expectations were manipulated in an orthogonal fashion, attentional selection was contingent upon perceptual expectation of visual stimuli. The attentional modulation of the anterior N1 component, which is thought to reflect top-down attentional orienting in the dorsal fronto-parietal network, was only observed for stimuli with expected orientation, whereas it was absent for unexpected stimuli. The attentional capture by task-relevant stimuli reflected in the N1pc component did not interact with perceptual expectations. However, expectations *additively* influenced early lateral posterior responses, consistent with a perceptual sharpening of the expected input. These findings suggest that, within 200 ms post-stimulus onset, attention and perceptual expectations may influence visual processing in a dissociable and interactive manner, where top-down attentional engagement is dependent on probabilistic feature expectations.

## Electronic supplementary material

Supplementary Information
