# Supplementary material for: Attentional gain is modulated by probabilistic feature expectations in a spatial cueing task: ERP evidence
Source: Sci Rep. 2018 Jan 8;8:54. doi: 10.1038/s41598-017-18347-1 (PMC5758810; doi:10.1038/s41598-017-18347-1)
Supplement: Supplementary file 1 — Supplementary Information [file 41598_2017_18347_MOESM1_ESM.pdf]

Attentional gain is modulated by probabilistic feature expectations in a spatial cueing task: ERP evidence

Supplementary information

Anna Marzecová[1, 2], Antonio Schettino[3], Andreas Widmann[1], Iria SanMiguel[4, 5, 6], Sonja A. Kotz[7], Erich Schröger[1]

[1]Institute of Psychology, University of Leipzig, Leipzig, Germany

[2]Department of Experimental and Applied Psychology, Vrije Universiteit Brussel, Brussels, Belgium

[3]Department of Experimental-Clinical and Health Psychology, Ghent University, Ghent, Belgium

[4]Brainlab-Cognitive Neuroscience Research Group, Department of Clinical Psychology and Psychobiology, University of Barcelona, Barcelona, Spain

[5]Institute of Neurosciences, University of Barcelona, Barcelona, Spain

[6]Institut de Recerca Sant Joan de Déu (IR-SJD), Barcelona, Spain

[7]Faculty of Psychology and Neuroscience, Department of Neuropsychology & Psychopharmacology, Maastricht University, Maastricht, The Netherlands

Supplementary information

Effects of attention and prediction on the contralateral and ipsilateral P1. We additionally explored effects of attention and prediction and their interaction on the amplitude of the contralateral (80–110 ms) and ipsilateral P1 (122–168 ms) component. A two-way rANOVA on amplitude values of the contralateral P1 component (80-110 ms) in the cluster of contralateral posterior electrodes (‘PO7/8c’, ‘P7/8c’, and ‘P5/6c’) did not reveal significant effects of attention ($F(1,17) = 0.74$, $p = .400$, $\eta^2_G = .006$), prediction ($F(1,17) = 2.80$, $p = .113$, $\eta^2_G = .018$), or their interaction ($F(1,17) = 1.25$, $p = .280$, $\eta^2_G = .012$). Bayesian analysis provided strong evidence in favour of the null model compared to the full attention × prediction model, $BF_{10} = 0.06 \pm 3.97\%$. Anecdotal evidence for the null model against the model with the main effect of prediction (the strongest model) was obtained, $BF_{10} = 0.50 \pm 1.37\%$ (see Table S1 for additional data).

The ipsilateral P1 (122–168 ms) amplitude in the cluster of ipsilateral posterior electrodes (‘PO7/8i’, ‘P7/8i’, and ‘P5/6i’) was more positive for attended ($M = 2.20$ µV, $SD = 1.88$) relative to unattended stimuli ($M = 1.38$ µV, $SD = 1.78$; see Fig. 2). A two-way rANOVA showed a significant main effect of attention ($F(1,17) = 9.53$, $p = .006$, $\eta^2_G = .046$), whereas the main effect of prediction and the attention × prediction interaction did not reach significance (Fs < 0.65, ps $> .43$, $\eta^2_G < .001$). Bayesian analysis indicated that a model which included attention as a single factor ought to be strongly preferred over the null model, $BF_{10} = 158.77 \pm 1.10\%$. This model was also 17.53 times more likely than the full model (see Table 1). To conclude, attention seem to have influenced the ipsilateral P1 only, while prediction did not reliably influence the ipsilateral or the contralateral P1.

Effects of attention and prediction at later processing stages? At later processing stages, effects of expectations may be characterised by a larger fronto-central P3 response in response to unexpected stimuli P3a (or novelty P3)[1,2]. Unexpected but task-relevant deviant stimuli

usually elicit larger P3b responses that are associated with updating of the internal representations[3,4]. Therefore, we additionally assessed effects of attention and prediction on the amplitude of P3a and P3b components. The P3a component was assessed in the cluster of fronto-central midline electrodes in the time window of 248–278 ms. The analysis of the parietal P3b component was conducted in the cluster of three midline parietal-occipital electrodes in the time window of 330–390 ms. To analyse modulations of these two components, rANOVAs were performed.

Two-way rANOVAs including factors attention (attended, unattended) and prediction (predicted, unpredicted) were performed to analyse the amplitudes of the P3a and P3b components at fronto-central electrodes (‘Fz’, ‘FCz’, and ‘Cz’) and parieto-occipital midline electrodes (‘CPz’, ‘Pz’, and ‘POz’) for the P3a and the P3b, respectively. In the time window of the P3a component (248–278 ms), amplitudes were more positive for unattended (M = +6.46 μV, SD = 3.71 μV) than for attended (M = +4.90 μV, SD = 3.83 μV) stimuli. However, a 2 x 2 rANOVA on Box-Cox transformed data did not reveal any significant main effects or interaction, all Fs < 2.28, ps > .149, $\eta^2_G < .044$. Bayesian analysis of the Box-Cox transformed data indicated that the model including the main effect of attention was weakly preferred over the null model, BF_{10} = 2.69 ±1.46%. This model was also 20.32 more likely than the full model (see Table S1 for additional data).

In the time window of the P3b component (330–390 ms), a 2 x 2 rANOVA on transformed data showed a significant main effect of attention, $F(1,17) = 22.86$, p < .001, $\eta^2_G = .204$. Attended stimuli elicited larger positivity compared to unattended stimuli (attended condition: M = 9.65 μV, SD = 5.18 μV, unattended condition: M = 5.28 μV, SD = 3.34 μV). The main effect of prediction and the attention x prediction interaction were not significant (Fs < 0.70, ps > .42, $\eta^2_G < .001$). Bayesian analysis indicated that the model with a single main effect of attention should be preferred over the null model, BF_{10} = 20503053 ±1.35%. This model was

23 times more likely than the full model (see Table S1). Therefore, in the current study, only the evidence for a modulation of P3b by attention was found. P3b component has been related to evaluation of task-relevant stimulus dimensions and motivational relevance of stimuli, and its amplitude and latency is therefore strongly related to task performance[2,3]. As the manipulation of expectations in the current study was related to task-irrelevant features of the stimulus, they may not have influenced the later processing stages reflected in the P3b component, reflecting task-relevant processing.

References

1.Friedman, D., Cycowicz, Y. M. and Gaeta, H. The novelty P3: an event-related brain potential (ERP) sign of the brain's evaluation of novelty. *Neurosci. Biobehav. Rev.* **25,** 355-73 (2001).

2.Nieuwenhuis, S., De Geus, E. J. and Aston-Jones, G. The anatomical and functional relationship between the P3 and autonomic components of the orienting response. *Psychophysiology* **48,** 162-75 (2011).

3.Polich, J. Updating P300: an integrative theory of P3a and P3b. *Clin. Neurophysiol.* **118,** 2128-48 (2007).

4.Kotz, S. A., Stockert, A. and Schwartze, M. Cerebellum, temporal predictability and the updating of a mental model. *Phil. Trans. R. Soc. B Biol. Sci.* **369,** 20130403 (2014).

Table S1. Bayes factors (BF_{10}) and percentage of proportional errors (% pe) for each model of interest in the auxiliary analyses, obtained by using JZS priors with a scaling factor of r = 0.707 (see Methods section for more details). The models with the best explanatory power are highlighted in bold.

Component	Model	BF_{10}	± % pe
contralateral P1 (80-110 ms)	Att	0.25	1.4
	Pred	0.50	1.37
	Att+Pred	0.12	1.7
	Att+Pred+Att*Pred	0.06	3.97
ipsilateral P1 (122-168 ms)	**Att**	**158.77**	**1.1**
	Pred	0.20	0.88
	Att+Pred	38.35	9.92
	Att+Pred+Att*Pred	9.05	2.92
P3a (248-278 ms)	Att	2.69	1.46
	Pred	0.19	1.56
	Att+Pred	0.51	1.05
	Att+Pred+Att*Pred	0.13	2.47
P3b (248-278 ms)	**Att**	**20503053**	**1.53**
	Pred	0.19	1.65
	Att+Pred	3813782	1.25
	Att+Pred+Att*Pred	1050522	9.23
